# Behavioral Activation (BA) in the Management of Depression in an Adolescent with Down Syndrome in Dubai

**DOI:** 10.1155/2021/7112034

**Published:** 2021-09-18

**Authors:** Sidra Shadan, Sarah Almarzooqi, Meshal A. Sultan

**Affiliations:** ^1^College of Medicine, Mohammed Bin Rashid University of Medicine and Health Sciences, Dubai, UAE; ^2^Mental Health Centre of Excellence, Al Jalila Children's Speciality Hospital, Dubai, UAE

## Abstract

Depression has been commonly treated with psychotherapy and/or pharmacotherapy for several decades. Ongoing research in the field has suggested promise for behavioral activation (BA), a form of psychotherapeutic intervention, as a means of increasing engagement in adaptive activities and developing skills to counter avoidance in individuals suffering from depression. In this case report, we present the treatment course of BA for an adolescent with Down syndrome (DS), presenting with depression. A multidisciplinary approach was utilized in developing a personalized management plan for the patient since the initial presentation. Sessions at the outpatient psychiatry clinic alternated between in-person visits and virtual ones, due to circumstances associated with physical distancing with the COVID-19 pandemic. Parents were included as integral parts of the management plan, and education, strategic implementation of BA, and barriers to care were discussed extensively to support the adolescent through the course of her treatment. Within 6 weeks of introducing BA, positive outcomes were noted in the patient, with the resolution of her clinical depression. In this report, we discuss BA further as a potentially effective therapeutic approach to the treatment of depressive symptoms in children and adolescents with DS and intellectual disabilities.

## 1. Background

Affecting more than 264 million people [1], depression is a leading cause of illness and disability globally and a major contributor to the overall burden of disease [2]. Characterized with a persistent low mood and a loss of interest in activities, depression often has a debilitating impact on an individual's daily functioning [3]. Among the younger demographic, a meta-analysis conducted in 2006 suggested that the overall prevalence of depression worldwide is at 2.8% for children under the age of 13 years and 5.7% for adolescents between the ages of 13 and 18 years [4]. Studying prevalence within the United Arab Emirates (UAE), Razzak and authors further conducted a systematic review to analyze studies published between 2007 and 2017 and determined depression prevalence scores to be 12.5% to 28.6% [3]. Although they attributed the large range to the widely varied study populations over the 14 analyzed articles, it was concluded that further research pertaining to specific populations was required in the region [3].

While depression is a leading cause of disease burden globally, Down syndrome (DS), on the other hand, is the most common genetic abnormality leading to intellectual disability worldwide [5]. The global incidence of DS has been reported as 1 per 1,000 births [5]. Caused by an additional partial or complete third copy of chromosome 21, patients with DS often present with varying severities of learning disability, growth retardation, and other medical abnormalities [6]. With regards to the UAE, a study conducted by Murthy and authors during a 5-year period between 1999 and 2003, including a total of 63,398 newborn babies in Dubai, determined the overall incidence of DS in this population to be 2.2 per 1,000 live births [7]. The authors reported that higher incidence rates of DS, compared to the global rates, may be due to advanced maternal age—with mothers bearing children until their 50s, multiparity, and the practice of consanguineous marriages [5, 7]. They further indicated that accessible genetic counselling with the wide-scale implementation of prenantal diagnostic services, and antenatal screening programs may reduce the psychological impact of DS on families in this community [7].

Children with DS are estimated to have comorbid neurobehavioral and psychiatric disorders, not specified for depression, at a prevalence rate of 18% to 38% [8–11]. According to a literature analysis conducted by Walker and authors, although not exclusively studying the younger demographic, the prevalence of depression specifically among those with DS ranged from 0 to 11.1% over the 30 analyzed articles [12]. Presently, research in the form of case reports and case series have showcased data available from small sample sizes including those from schools, communities, and clinics [13]. Although these studies have described psychiatric and behavioral disorders in children and adolescents with DS, the terminology, diagnostic criteria, and the implemented study design vary widely [13]. Furthermore, current literature fails to consider the complex circumstances in which these disorders may arise. As a result, very little is known about how children with DS presenting with psychiatric conditions fare with regard to associated medical conditions, developmental characteristics, or treatment management and subsequent outcomes [13].

In comparison with other causes of intellectual disability, in 1992, Collacott and authors suggested that individuals with DS were particularly prone and vulnerable to depression [12, 14]. Their findings indicated that a high incidence of depression, along with evidence of weak detection and inadequate treatment, may have contributed to a greater burden of suffering for individuals with DS [12, 14]. Contrary to their work, however, a recent study conducted by Mantry and authors in 2008 proposed that DS is often accompanied by many psychiatric disorders and that earlier suggestions of an increased prevalence of depression specifically are not supported in DS—although adequate statistical analysis was not conducted due to low participation in the study [12, 15].

Despite of there being debate on which neurobehavorial or psychiatric disorders are seen predominantly in DS, it is known that individuals with DS are exposed to high levels of environmental stressors and due to their vulnerabilities may be susceptible to the development of depression [12]. In their study, Walker and authors analyzed the potential risk factors for the development of depression as either general risk factors or those more specific to DS [12]. General risk factors included smaller brain volumes [16], lower intelligence quotient (IQ) levels [17], and stressful life events accompanied with increased awareness of being different from the general population, social withdrawal [18], female gender [19], and family history [20]. On the other hand, risk factors for the development of depression more specific in the setting of DS included smaller hippocampal volumes associated with relapse of depressive symptoms [21], changes in neurotransmitter pathways [22], neuropsychological deficits pertaining to language and memory [23], attachment behavior [24], and associated somatic disorders including physical disabilities and hypothyroidism [25, 26].

Individuals with DS may frequently also experience obsessions, compulsions, and severe generalized anxiety as a result of major depression [20]. Some may further exhibit psychotic or catatonic-like characteristics, making the detection and treatment of their depression more difficult [20, 27]. Medical conditions such as obstructive sleep apnea (OSA) should be ruled out in individuals presenting with immense fatigue and daytime somnolence, as OSA may mimic depressive symptoms [20, 28].

The diagnosis of depression in DS is thus based heavily upon observable characteristics, and therefore, the use of modified diagnostic criteria is often advised [12]. Although several common treatments such as psychotherapy—in the form of cognitive behavioral therapy (CBT) and interpersonal therapy (IPT)—and pharmacotherapy—with prescribed selective serotonin reuptake inhibitors (SSRIs) such as fluoxetine and citalopram [29]—seem effective, there is evidence that depression in DS is undertreated [14]. The American Psychiatric Association and the American Academy of Child and Adolescent Psychiatry have recommended that psychotherapy should always be incorporated, as either the sole management in milder depression or in combination with medications in those with moderate to severe depression [12, 29]. Numerous studies further present the effectiveness of CBT in individuals with mild intellectual disabilities and depression [30, 31].

Alongside CBT and IPT, behavioral activation (BA), a form of psychotherapeutic intervention, has been adapted as means to treat depression, among other mental health disorders, for the last several decades [32]. Based on the behavioral models of Lewinsohn in the 1970s, it primarily stems from the notion that depression is a consequence of a lack of positive reinforcement, as depressed individuals not only enjoy daily activities less than baseline but also engage less frequently in pleasurable activities [32, 33]. With numerous component techniques of BA—for example, activity monitoring and scheduling, skills training, and procedures targeting avoidance—it aims to bring about behavioral change in the individual's life [32, 34, 35]. Through BA, individuals learn to recognize the connection between their activities and its impact on their daily mood and use this understanding to plan activities in a manner that creates positive reinforcement and counters avoidance behaviours [32].

In this case report, we describe the course of management of BA for an adolescent, diagnosed with Down syndrome, treated in Dubai, UAE. To protect the privacy of the patient and maintain confidentiality, we will use the pseudonym of Amira Faris throughout this report.

## 2. Case Presentation

“Amira Faris” is a 12-year-old girl of Arabic origin. She has Down syndrome and intellectual challenges. Amira lives with both parents in Dubai and has five older siblings. She is enrolled in a special education center; however, it got closed six months ago in the context of the COVID-19 pandemic.

Amira was assessed at the outpatient clinic by a consultant child and adolescent psychiatrist. She presented with her parents due to concerns related to irritability, excessive crying, and sleep disturbance over the past 3 months. Along with these symptoms, she developed excessive fearfulness, was noted to speak without a clear reason, and pointed randomly saying “bugs” and “ghost.” Furthermore, her parents reported a decline in appetite, excessive tiredness, poor concentration, and Amira's decreased interest in communicating with them. Since the closure of the special education center, she did not have a structure during her day and spent most of the time at home. On some occasions, she went to the park; however, expressed feeling tired, and therefore, returned home shortly. At baseline, she is described as joyful, and she likes to sing. Additionally, she was noted to enjoy the time she spent with her classmates at the special education center. Prior to the changes in her condition, she used to speak in two to three-word phrases and was able to write and read simple words.

Within a few weeks of the emergence of her symptoms, she was assessed by a psychiatrist in the community who indicated that she had auditory and visual hallucinations, for which the psychiatrist prescribed antipsychotic medication, aripiprazole. Her visual and auditory perceptual disturbance resolved within one month of taking the medication, and then the medication was gradually discontinued within two weeks. However, her depressive symptoms persisted.

From a medical perspective, Amira has hypothyroidism, and she has been prescribed thyroxin. She follows regularly at the endocrinology clinic, and her recent thyroid function tests were within normal limits. She had cardiac surgery for atrioventricular septal defect (AVSD) during infancy. She has been following at the cardiology clinic twice per year. Her medical condition has been stable in this regard with no reported complications. Amira has myopia and wears glasses to correct her vision.

At the initial evaluation, Amira's engagement with the interviewer and with her parents was minimal. She appeared tired and was generally slow to respond. Her facial expressions were blunted and she came across as not interested in conversation. She required prompts to answer questions and only spoke in a few single words. The psychiatric assessment was based on the Diagnostic and Statistical Manual of Mental Disorders Fifth Edition (DSM-5) and revealed that her presentation was in keeping with major depressive disorder. The abrupt suspension in going to the special education center, lack of interaction with peers, and disruption of her daily routine was likely to have contributed to the development of her symptoms.

Her follow-ups at the outpatient psychiatry clinic alternated between virtual and in person visits, with the frequency being two weeks initially and then monthly. The management plan included starting with the implementation of a behavioral activation program. Her current daily activities were recorded, as well as her associated mood. Education was provided about the association between the nature of activities and its impact on the individual's emotions. With the involvement of her parents, a plan for scheduling daily activities was set. Education was provided about taking Amira's values into consideration during activity planning. Furthermore, the importance of scheduling activities associated with positive emotions was highlighted. Barriers of following through with scheduled plans were discussed and solutions were generated.

Amira's new daily routine included meeting with an occupational therapist at home to assist her with activities of daily living, as well as going for physiotherapy sessions. Additionally, her parents scheduled time for indoor activities, for instance, playing with dolls, and outdoor activities, such as going to the swimming pool. After two months of her initial assessment, the special education center gradually reopened, and she started to attend two days per week.

Within four weeks of implementing the BA plan, a significant improvement was noted in Amira's mood and daily functioning. The Mood and Feelings Questionnaire (MFQ) completed by her parents reflected a decline in total score from 39 at her initial visit to 21 at her 4-week visit. A score of 21 or more on the MFQ is suggestive of clinical depression [36]. The Screen for Child Anxiety Related Disorders (SCARED) completed by her parents reflected a decline in total score from 24 at her initial visit to 15 at her 4-week visit. A score of 25 or more on the SCARED is suggestive of clinical anxiety [37]. The depressive and anxiety symptoms improved further at six weeks of starting the program, as the MFQ and SCARED total scores reported by Amira's parents decreased to 13 and 10, respectively. By the 6-week follow-up, Amira was no longer meeting the criteria for clinical depression.

In addition to the changes that were reported in the rating scales, Amira's parents noted that she had become more interested in spending time with them. Additionally, she seemed to enjoy the activities that were included in her daily schedule. Her sleep became continuous for nine to ten hours per night. She started to eat by herself, and her appetite improved. Her energy levels, as well as her concentration, have improved. At six weeks of implementing the behavioral activation program, her functioning was described to be back to baseline. At the clinic, Amira came across as having an euthymic mood. She engaged in answering simple questions with appropriate brief phrases. She made requests politely, for instance, by pointing at a drawing on the wall and asking to get a similar one. She colored the drawing and shared her work with her parents and the interviewer. Amira's progress was maintained throughout her subsequent visits until her most recent follow-up at three months of implementing the behavioral activation program.

## 3. Discussion

An adolescent with Down syndrome and intellectual challenges, presenting with depressive symptoms, was presented in this report. Over the course of the patient's management, a significant improvement was noted in her daily functioning, engagement with parents, and overall mood. The result of this case report is consistent with current studies which demonstrate a positive effect on the mood of children and adolescents, along with reduction in depressive symptoms, with the implementation of a BA program [38].

A literature review revealed that the most common symptoms of depression in individuals with DS were loss of interest, decreased appetite, sleep disorder, and anxiety [12]. Hallucinations were reported in 46% of the sample in one study and in 5% of the sample in another study [12]. It can be difficult to differentiate between regression and depression due to the overlap in their presentation. A retrospective study of 30 individuals with DS who experienced acute regression highlighted the following symptoms: sadness (30%), anxiety (10%), delusions (13%), and sleep disturbance (10%) [39]. The case that we have presented likely had major depressive disorder since she has met the DSM-5 criteria for this disorder.

The prevalence of autism spectrum disorder (ASD) in individuals with DS is higher than the general population and ranges from 1 to 15% [40, 41]. Furthermore, children with ASD experience various cognitive challenges, which include cognitive inflexibility [42]. The presence of comorbid ASD; therefore, negatively impacts their ability to adjust to changes in routine. In the case we have presented, screening questions did not suggest the presence of ASD; however, we did not conduct a comprehensive evaluation in this regard.

In the assessment of individuals with DS, medical conditions that may mimic symptoms of depression need to be ruled out, for instance, hypothyroidism and dementia [43]. Furthermore, screening for various medical conditions that may impact the individual's quality of life is recommended. The required evaluations include echocardiogram, ophthalmological assessment, and hearing assessment [43]. Additionally, monitoring for various conditions is recommended, including obesity, coeliac disease, arthritis, diabetes mellitus, and seizures [43]. Parental support contributes positively to improvement in quality of life of individuals with DS [43]. A study of adults, with mild to moderate intellectual disability, has revealed efficacy of cognitive behavioral therapy (CBT) for management of depression [31]. However, additional studies are required to generalize findings to individuals with DS [31].

A 2017 meta-analysis conducted by Martin and Oliver of four randomized control trials (RCTs) reported a large effect size of BA for pediatric population and demonstrated a statistically significant difference in favor of BA in comparison to the control groups [38]. However, two limitations were discussed in the article. First, sample sizes were small, with the largest participant size of 185 individuals analyzed in one of the studies. Second, the analysis across the four RCTs was conducted for different control groups as the following: two studies offered no active intervention for the control group; one made referrals to mental health services and the last study provided active treatment in the form of evidence-based practice for depression. Despite of the reported limitations in the meta-analysis, scores in the Children's Depression Rating Scale-Revised (CDRS-R) reported a reduction in the depression of the BA intervention groups across the 4 RCTs [38].

A literature review revealed that studies on psychotherapy among individuals with intellectual disability focused on behavioral strategies and skill acquisition [44]. Individuals with intellectual disability present with deficits in self-motivation and planning. Therefore, including a significant other in psychotherapy can support the individual in implementing the lifestyle changes [45].

Although BA had a role in positively impacting the adolescent presented in our case report, other psychosocial factors may have also contributed to enhancing the outcome. It is key to recognize that the onset of her depressive symptoms roughly emerged after the closing of the special education center. However, two months into the BA therapy, the center reopened, allowing the patient to resume and rejoin her peers. Additionally, the assistance provided by the occupational therapist may have contributed to her improvement, along with the strengthened relationship with her parents due to more active time being spent together as a family. Such psychosocial factors may have contributed to the resolution of her depressive symptoms; however, the role of BA in having a significant positive impact on the patient cannot be overlooked.

Current neurodevelopmental data suggests that adolescents are prone to increased sensitivity to social or environmental stressors, difficulties in processing rewards, and have the tendency to avoid emotional stimuli [46, 47]. Studies on psychological disengagement suggest that based on the available neurodevelopmental data, treatment for depression in the young may need to address the adolescents' ability to not only experience and respond to reward but also counter avoidance [46]. As BA focuses on these concepts, by addressing environmental and social stressors, encouraging interaction with positive reinforcers, and developing strategies to increase engagement and overcome barriers, it may prove to be an effective approach in the management of depressed children and adolescents [48].

Both cognitive behavioral therapy and interpersonal therapy, adapted and modified for the use in a younger demographic, have showcased efficacy in the treatment of depression [38]. However, the effect size in current literature remains small [49], and residual symptoms of depression remain taxing on some individuals [50]. Despite undergoing psychotherapy and/or pharmacotherapy, some youth do not achieve remission of depression, and relapse is seen postintervention [51, 52]. Furthermore, important subgroups within children and adolescents, such as those exposed to adversity in early stages of life, and those who are less open to partake in “talking therapy,” demonstrated poor response to existing treatments [53–55].

According to Martin and Oliver, when considering if BA is an appropriate intervention for children and youth for addressing their mental health difficulties, four considerations of an intervention must be determined: (1) the intervention is appropriate for the developmental stage of the individual (for example, someone with intellectual disabilities may find it difficult to partake in complex abstract thinking but may find it easier to follow through with behavioral changes) [56]; (2) clinically, the intervention can address frequent comorbid disorders, such as depression and anxiety, and thus, maximize impact for a range of presentations [57]; (3) the intervention is culturally sensitive to values and understanding of distress, and thus, can be implemented widely in diverse and multicultural settings [58]; and (4) the intervention is scalable, requiring minimal resources (for example, nonspecialist delivery/therapy) to reach most within a population [59]. Behavioral activation meets all these considerations being feasible for adolescents targeting behavior through a simple, concrete approach. It is highly personalized to meet individual needs, taking cultural values and individual priorities into consideration. It is proved to be an effective treatment for depression having been extensively studied in adults [60, 61], can be delivered by non-specialists, and is not clinically inferior to traditional psychotherapy, namely, CBT and IPT [61–63].

Research into BA for children and adolescents is an emerging field, with current literature supporting its impact on reducing and/or treating depression. However, ongoing research must be conducted to include larger sample sizes for greater effect size and power [38, 55]. Throughout our literature search, studies that implemented a specific BA program in individuals with DS presenting with depression were limited. Given the success and positive outcome seen in the adolescent of the presented case report, further research is recommended on not only the prevalence of different psychiatric disorders in individuals with DS and other intellectual disabilities but also comparisons on effective treatment modalities for this subgroup of children and adolescents.

## 4. Conclusion

Throughout the decades, BA has revealed significant promise for the treatment of depression and other psychiatric disorders. This case report demonstrated that BA was effective in the management of depression in an adolescent with DS. Furthermore, the positive effects persisted at three months of follow-up. Parental support, resuming daily routine, and being physically healthy have likely contributed positively towards recovery. Additionally, this article has highlighted the marked impact of abrupt changes in lifestyle and social interactions especially on individuals with intellectual challenges.

Although treatment recommendations and guidelines for individuals experiencing depression differ between adults and young people, given the extensive research supporting the use of BA with adults, it would be important to further investigate its feasibility and impact in treating the younger demographic, especially in subgroups who present with underlying medical conditions and may benefit with alternative approaches to the treatment of their depression. Thus, innovative treatment, in the form of BA or the development of new therapeutic approaches, remains of utmost importance in this field.

## Data Availability

Data used to support the findings of this study are included within the article.

## References

[1] James S. L., Abate D., Abate K. H. (2018). Global, regional, and national incidence, prevalence, and years lived with disability for 354 diseases and injuries for 195 countries and territories, 1990-2017: a systematic analysis for the Global Burden of Disease Study 2017. *The Lancet*.

[2] Erskine H., Moffitt T. E., Copeland W. (2015). A heavy burden on young minds: the global burden of mental and substance use disorders in children and youth. *Psychological Medicine*.

[3] Abdul Razzak H., Harbi A., Ahli S. (2019). Depression: prevalence and associated risk factors in the United Arab Emirates. *Oman Medical Journal*.

[4] Jane Costello E., Erkanli A., Angold A. (2006). Is there an epidemic of child or adolescent depression?. *Journal of Child Psychology and Psychiatry*.

[5] Al-Biltagi M. (2015). Down syndrome from epidemiologic point of view. *EC Paediatrics*.

[6] Akhtar F., Bokhari S. R. A. (2021). *Down Syndrome. [Updated 2021 Mar 30]. In: StatPearls [Internet]*.

[7] Murthy S. K., Malhotra A. K., Mani S. (2006). Incidence of Down syndrome in Dubai, UAE. *Medical Principles and Practice*.

[8] Gath A., Gumley D. (1986). Behaviour problems in retarded children with special reference to Down’s syndrome. *The British Journal of Psychiatry*.

[9] Gillberg C., Persson E., Grufman M., Themner U. (1986). Psychiatric disorders in mildly and severely mentally retarded urban children and adolescents: epidemiological aspects. *The British Journal of Psychiatry*.

[10] Myers B., Pueschel S. M. (1991). Psychiatric Disorders in persons with Down syndrome. *The Journal of Nervous and Mental Disease*.

[11] Dykens E. M., Shah B., Sagun J., Beck T., King B. H. (2002). Maladaptive behaviour in children and adolescents with Down’s syndrome. *Journal of Intellectual Disability Research*.

[12] Walker J. C., Dosen A., Buitelaar J. K., Janzing J. G. E. (2011). Depression in Down syndrome: a review of the literature. *Research in Developmental Disabilities*.

[13] Capone G., Goyal P., Ares W., Lannigan E. (2006). Neurobehavioral disorders in children, adolescents, and young adults with Down syndrome. *American Journal of Medical Genetics Part C: Seminars in Medical Genetics*.

[14] Collacott R. A., Cooper S. A., McGrother C. (1992). Differential rates of psychiatric disorders in adults with Down’s syndrome compared with other mentally handicapped adults. *British Journal of Psychiatry*.

[15] Mantry D., Cooper S. A., Smiley E. (2008). The prevalence and incidence of mental ill-health in adults with Down syndrome. *Journal of Intellectual Disability Research*.

[16] Thase M. E. (2005). *Mood Disorders: Neurobiology Kaplan and Sadock’s Comprehensive Textbook of Psychiatry*.

[17] Zammit S., Allebeck P., David A. S. (2004). A longitudinal study of premorbid IQ score and risk of developing schizophrenia, bipolar disorder, severe depression, and other nonaffective psychoses. *Archives of General Psychiatry*.

[18] Ailey S. H., Miller A. M., Heller T., Smith E. V. (2006). Evaluating an interpersonal model of depression among adults with Down syndrome. *Research and Theory for Nursing Practice*.

[19] Tsakanikos E., Bouras N., Sturmey P., Holt G. (2006). Psychiatric co-morbidity and gender differences in intellectual disability. *Journal of Intellectual Disability Research*.

[20] Myers B. A., Pueschel S. M. (1995). Major depression in a small group of adults with Down syndrome. *Research in Developmental Disabilities*.

[21] Coryell W., Nopoulos P., Drevets W., Wilson T., Andreasen N. C. (2005). Subgenual prefrontal cortex volumes in major depressive disorder and schizophrenia: diagnostic specificity and prognostic implications. *American Journal of Psychiatry*.

[22] Whittle N., Sartori S. B., Dierssen M., Lubec G., Singewald N. (2007). Fetal Down syndrome brains exhibit aberrant levels of neurotransmitters critical for normal brain development. *Pediatrics*.

[23] Silverman W. (2007). Down syndrome: cognitive phenotype. *Mental Retardation and Developmental Disabilities Research Reviews*.

[24] Ganiban J., Barnett D., Cicchetti D. (2000). Negative reactivity and attachment: Down syndrome’s contribution to the attachment-temperament debate. *Development and Psychopathology*.

[25] Cooper S. A., Smiley E., Morrison J., Williamson A., Allan L. (2007). An epidemiological investigation of affective disorders with a population-based cohort of 1023 adults with intellectual disabilities. *Psychological Medicine*.

[26] Joffe R. T. (2006). Is the thyroid still important in major depression?. *Journal of Psychiatry and Neuroscience*.

[27] McGuire D. E., Chicoine B. A. (1996). Depressive disorders in adults with Down syndrome. *Habilitative Mental Healthcare Newsletter*.

[28] Resta O., Foschino Barbaro M., Giliberti T. (2003). Sleep related breathing disorders in adults with Down syndrome. *Down's Syndrome, Research and Practice*.

[29] Clark M. S., Jansen K. L., Cloy J. A. (2012). Treatment of childhood and adolescent depression. *American Family Physician*.

[30] Lindsay W. R., Howells L., Pitcaithly D. (1993). Cognitive therapy for depression with individuals with intellectual disabilities. *British Journal of Medical Psychology*.

[31] McCabe M. P., McGillivray J. A., Newton D. C. (2006). Effectiveness of treatment programmes for depression among adults with mild/moderate intellectual disability. *Journal of Intellectual Disability Research*.

[32] Kanter J. W., Manos R. C., Bowe W. M., Baruch D. E., Busch A. M., Rusch L. C. (2010). What is behavioral activation?: a review of the empirical literature. *Clinical Psychology Review*.

[33] Lewinsohn P., Friedman R. J., Katz M. M. (1974). A behavioral approach to depression. *Psychology of Depression: Contemporary Theory and Research*.

[34] Lewinsohn P. M., Biglan A., Zeiss A. M., Davidson P. O. (1976). Behavioral treatment for depression. *Behavioral Management of Anxiety, Depression and Pain*.

[35] Martell C. R., Addis M. E., Jacobson N. S. (2001). *Depression in Context: Strategies for Guided Action*.

[36] Wood A., Kroll L., Moore A., Harrington R. (1995). Properties of the mood and feelings questionnaire in adolescent psychiatric outpatients: a research note. *Journal of Child Psychology and Psychiatry*.

[37] Birmaher B., Brent D. A., Chiappetta L., Bridge J., Monga S., Baugher M. (1999). Psychometric properties of the screen for child anxiety related emotional disorders (SCARED): a replication study. *Journal of the American Academy of Child and Adolescent Psychiatry*.

[38] Martin F., Oliver T. (2019). Behavioral activation for children and adolescents: a systematic review of progress and promise. *European Child & Adolescent Psychiatry*.

[39] Mircher C., Cieuta-Walti C., Marey I. (2017). Acute regression in young people with Down syndrome. *Brain Sciences*.

[40] Reilly C. (2009). Autism spectrum disorders in Down syndrome: a review. *Research in Autism Spectrum Disorders*.

[41] DiGuiseppi C., Hepburn S., Davis J. M. (2010). Screening for autism spectrum disorders in children with Down syndrome: population prevalence and screening test characteristics. *Journal of Developmental and Behavioral Pediatrics: JDBP*.

[42] Granader Y., Wallace G. L., Hardy K. K. (2014). Characterizing the factor structure of parent reported executive function in autism spectrum disorders: the impact of cognitive inflexibility. *Journal of Autism and Developmental Disorders*.

[43] Roizen N. J., Patterson D. (2003). Down's syndrome. *The Lancet*.

[44] Beail N. (2003). What works for people with mental retardation? Critical commentary on cognitive–behavioral and psychodynamic psychotherapy research. *Mental Retardation*.

[45] Jahoda A., Melville C. A., Pert C. (2015). A feasibility study of behavioural activation for depressive symptoms in adults with intellectual disabilities. *Journal of Intellectual Disability Research*.

[46] Forbes E. E. (2009). Where's the Fun in That? Broadening the Focus on Reward Function in Depression. *Biological Psychiatry*.

[47] Hare T. A., Tottenham N., Galván A., Voss H. U., Glover G. H., Casey B. J. (2008). Biological substrates of emotional reactivity and regulation in adolescence during an emotional go-nogo task. *Biological Psychiatry*.

[48] McMakin D. L., Olino T. M., Porta G. (2012). Anhedonia predicts poorer recovery among youth with selective serotonin reuptake inhibitor treatment-resistant depression. *Journal of the American Academy of Child & Adolescent Psychiatry*.

[49] Weisz J. R., McCarty C. A., Valeri S. M. (2006). Effects of psychotherapy for depression in children and adolescents: a meta-analysis. *Psychological Bulletin*.

[50] Kennard B., Silva S., Vitiello B. (2006). Remission and residual symptoms after short-term treatment in the Treatment of Adolescents with Depression Study (TADS). *Journal of the American Academy of Child & Adolescent Psychiatry*.

[51] Curry J., Silva S., Rohde P. (2011). Recovery and recurrence following treatment for adolescent major depression. *Archives of General Psychiatry*.

[52] Emslie G. J., Kennard B. D., Mayes T. L. (2008). Fluoxetine versus placebo in preventing relapse of major depression in children and adolescents. *The American Journal of Psychiatry*.

[53] Lewis C. C., Simons A. D., Nguyen L. J. (2010). Impact of childhood trauma on treatment outcome in the Treatment for Adolescents With Depression Study (TADS). *Journal of the American Academy of Child & Adolescent Psychiatry*.

[54] Nanni V., Uher R., Danese A. (2012). Childhood maltreatment predicts unfavorable course of illness and treatment outcome in depression: a meta-analysis. *American Journal of Psychiatry*.

[55] McCauley E., Gudmundsen G., Schloredt K. (2016). The adolescent behavioral activation program: adapting behavioral activation as a treatment for depression in adolescence. *Journal of Clinical Child & Adolescent Psychology*.

[56] Dimidjian S., McCauley E. (2016). Modular, scalable, and personalized: priorities for behavioral interventions for adolescent depression. *Clinical Psychology: Science and Practice*.

[57] Garber J., Weersing V. R. (2010). Comorbidity of anxiety and depression in youth: implications for treatment and prevention. *Clinical Psychology: Science and Practice*.

[58] Fernando S. (2014). *Mental Health Worldwide: Culture, Globalization and Development*.

[59] Lund C., Tomlinson M., De Silva M. (2012). PRIME: a programme to reduce the treatment gap for mental disorders in Five low- and middle-income countries. *PLoS Medicine*.

[60] Ekers D., Webster L., Van Straten A., Cuijpers P., Richards D., Gilbody S. (2014). Behavioural activation for depression; an update of meta-analysis of Effectiveness and sub group analysis. *PLoS One*.

[61] Richards D. A., Ekers D., McMillan D. (2016). Cost and outcome of behavioural activation versus cognitive behavioural therapy for depression (COBRA): a randomised, controlled, non-inferiority trial. *Lancet*.

[62] Kanter J., Puspitasari A. J., Santos M. M., Nagy G. A. (2012). Behavioural activation: history, evidence and promise. *The British Journal of Psychiatry*.

[63] Ekers D., Richards D., McMillan D., Bland J. M., Gilbody S. (2011). Behavioural activation delivered by the non-specialist: phase II randomised controlled trial. *The British Journal of Psychiatry*.

